# Making progress on identifying those who aren’t making progress: Using situational judgment tests to predict those at risk for remediation and attrition

**DOI:** 10.15694/mep.2018.0000275.1

**Published:** 2018-12-07

**Authors:** Aimee Gardner, Brian Dunkin

**Affiliations:** 1 Baylor College of Medicine; 2 Houston Methodist Hospital

**Keywords:** selection, situational judgment test, USMLE, remediation, attrition, surgery

## Abstract

This article was migrated. The article was marked as recommended.

Introduction

There is little evidence on tools that can be used to identify individuals most at risk for remediation and attrition in postgraduate surgical training. We explore the extent to which a situational judgment test (SJT) can predict which trainees are likely to require remediation or leave the program.

Methods

Postgraduate trainees in a single general surgery residency program in the United States completed a 50-item SJT. Data regarding remediation actions and attrition were retrieved from the program for the two years following completion of the assessment. United States Medical Licensing Examination (USMLE) scores were also included to examine their ability to predict remediation and attrition.

Results

Complete data was available from fifty-two of 56 (92%) residents in the program. Twenty-one percent (11/52) of residents required at least one remediation intervention within the one (for PGY5s) to two years after completing the SJT. Mann-Whitney U tests revealed a significant difference in SJT performance between those needing remediation versus those who did not require remediation, such that those requiring remediation performed worse on the assessment (p

Conclusions

These data demonstrate that SJTs can be created to effectively identify surgery residents most at risk for remediation across a two-year timeframe. These data provide an additional layer of validity evidence to support the role of SJTs in surgical education and align with other studies failing to find linkages between USMLE scores and residency performance criterion.

## Introduction

Modern surgical training is intense, fast-paced, and rigorous. To succeed in this environment, trainees must be able to quickly adapt to new environments, master new surgical techniques and technologies, and successfully work within dynamic teams. As a result of this unique environment, many postgraduate trainees fail to meet program expectations during their training and require additional focused interventions to get back on track. In the United States (U.S.), nearly one third of general surgery trainees require remediation during their five years of post-medical school training, with 25% of those remediated requiring more than one intervention ( Yaghoubian et al., 2012). Attrition rates are high as well, ranging from 20% - 30% and 62% of those who leave general surgery training go on to pursue a non-surgical specialty ( Longo et al., 2009; Yeo et al., 2017).

Many training programs have performed retrospective reviews of the reasons and types of remediation in their programs, in hopes of identifying early on those trainees at risk. Interestingly, despite the technical demands of surgical training, deficiency in technical skills is an uncommon reason for remediation and a rarely a reason for dismissal ( Khoushhal et al., 2017). When examining non-technical factors, Yaghoubian et al. (2012) reported a deficiency in medical knowledge as the most frequent reason for remediation in six US training programs (74%), while others have found that problems with professionalism and interpersonal skills were more frequent ( Bergen et al., 2000). Williams et al.(2009) examined the performance of surgical trainees in their program over a 30-year period and found that serious performance problems fell into three main categories: interpersonal skills, insufficient knowledge, and communication skills. Unfortunately, despite specific attempts at helping these trainees get back on track, 88% were refractory to remediation.

Given the high incidence of non-technical and non-cognitive issues requiring remediation in surgical training, it is not surprising that current methods of evaluating trainees or screening applicants for training fail to identify those at risk. In the U.S., traditional screening of residency applicants includes review of U.S. Medical Licensing Examination (USMLE) scores, medical school grades, letters of recommendation, personal statements, and unstructured interviews. However, these tools have been criticized not only for their inability to predict performance in residency, but also to identify those most at risk for remediation and attrition ( Gardner, Grantcharov, and Dunkin, 2018; McGaghie, Cohen and Wayne, 2011; Fryer et al., 2012; Stohl, Hueppchen, and Bienstock, 2010; Sutton et al., 2014; Zuckerman et al., 2018). Thus, there is a need to identify and develop tools that can predict who may be likely to struggle in surgical training.

In this study, we explore the extent to which a situational judgment test (SJT) can predict which trainees are likely to require remediation or leave the program. SJTs present candidates with a variety of situations they would likely to encounter if they were brought into the organization, and are asked to make judgments about the effectiveness of possible responses. SJTs have become widespread across organizations outside of medicine, likely because of the robust body of validity evidence that exists showing their ability to predict on-the-job performance ( Clevenger et al. 2001, Weekly and Ployhart 2001), reduce potential for adverse impact ( Hough, Oswald, and Ployhart, 2001), and provide applicants with realistic job previews ( Clevenger et al., 2001). Their multi-dimensional nature and capacity to measure the ability *to do* rather than the ability *to know* by placing candidates “in the situation” separates SJTs from more traditional, straight-forward assessments such as multiple-choice questions, short answer questions, and the like. As such, we explore the following research questions in this study:

1. *Can SJT scores be used to predict remediation and attrition in residency?*
2. *To what extent can SJT scores be used to complement examination scores in predicting resident remediation and attrition?*

## Methods

### Sample

Participants included postgraduate trainees in a single general surgery residency program in the United States. The program was chosen because of its relatively large number of residents and robust assessment database.

### Situational Judgment Test

A pool of 50 situational judgment test items were created by a team of MDs and PhDs. Semi-structured interviews with subject matter experts (SMEs) and review of historical resident performance data were used to identify which competencies were critical for residents to be successful in the program. Data from these interviews informed the development of SJT items, similar to prior studies ( Gardner and Dunkin 2018a; Lievens, Peeters, and Schollaert, 2008). Faculty SMEs central to the residency education program (Program Director, Associate Program Directors, members of the Clinical Competency Committee, etc.) reviewed all items and provided input regarding effectiveness of each item to inform which items ended up on the final tool and the scoring algorithm.

The final SJT was given to all postgraduate year (PGY) 1 - 5 residents at the beginning of the academic year. Results from the assessment were neither used for any developmental or assessment purpose nor were they shared with program faculty.

### Outcome Measures

Remediation data, defined as any documented action taken by program leadership to improve a resident deficit, were collected for the following two years. All remediation events were captured as dichotomous (yes/no) variables and categorized according to the Accreditation Council for Graduate Medical Education (ACGME) core competencies: medical knowledge, patient care, interpersonal and communication skills, professionalism, systems-based practice, and problem-based learning and improvement.

Resident attrition data were also collected. Residents who left the program prior to graduation for any reason (involuntary termination, transfer to another surgery program, transfer to another specialty, exiting medicine for another occupation, etc.) were included in the turnover group according to a dichotomous (yes/no) labeling.

### Analyses

Mann-Whitney U tests and *χ* ^2^ were used to compare the SJT score distributions for trainees who were marked positive for either remediation or attrition over the two-year time frame. As USMLE 1 scores are traditionally used as the primary initial screening tool among residency Program Directors in the United States (Program Director Survey, 2018), these data were also included as a potential predictor for remediation and turnover.

## Results

Complete data were available for 93% (52/56) of residents in the program. Residents consisted of categorical PGY1s (25%), PGY2s (19.2%), PGY3s (25%), PGY4s (17.3%), and PGY5s (13.5%).

**Figure 1.**
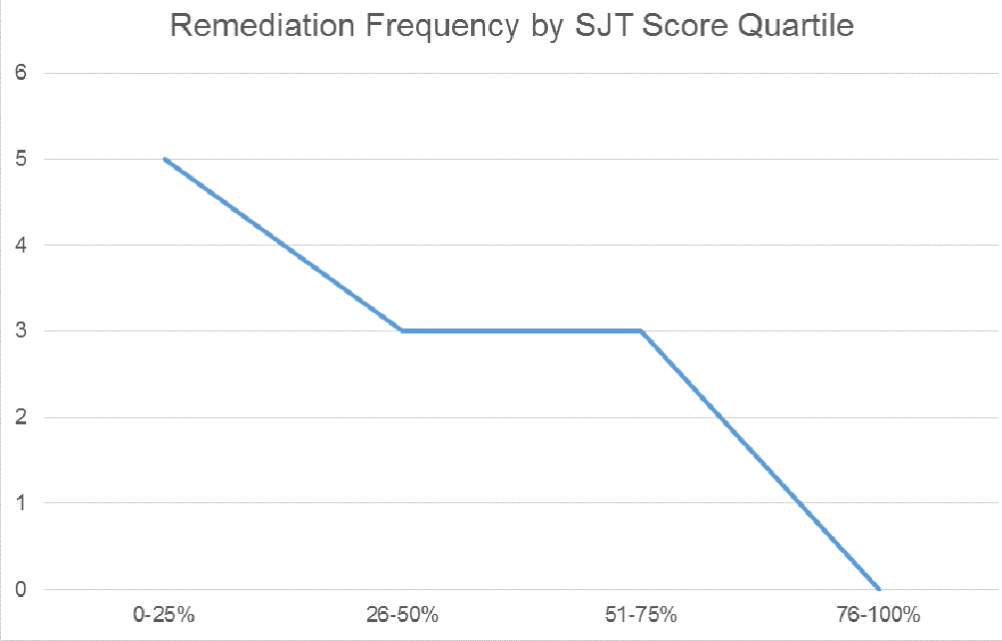
Remediation Frequency by SJT Score Quartile

Twenty-one percent (11/52) of residents required at least one remediation intervention within the one (for PGY5s) to two years after completing the SJT. The majority of these interventions were related to deficits in professionalism (56%), with the remaining related to decision-making (33%) or technical skills (11%). Mann-Whitney U tests revealed a significant difference in SJT performance between those needing remediation versus those who did not require remediation, such that those requiring remediation performed worse on the assessment (p = 0.04). Further exploration of remediation frequency by SJT score quartile reveals that trainees performing below top quartile of the SJT is associated with a 3-5 times higher likelihood of receiving a remediation intervention. Figure 1 displays these data based on performance on the SJT.

Eight percent (4/52) of residents left the program within the one to two years after completing the SJT. Two residents (PGY2 and PGY3) transferred to another general surgery program and the remaining two (both PGY1s) joined another medical specialty. There was not a significant difference in SJT scores between those who remained in the program 1-2 years after completing the assessment (p = 0.41).

Finally, we explore the relationship among USMLE1 scores and these variables. SJT and USMLE1 scores were not correlated. Additionally there were no differences in USMLE scores between the two groups in either remediation interventions (245 versus 244, p = 0.75) or attrition (243 versus 246, p = 0.67).

## Discussion

Identifying residents at risk for performance deficiencies and turnover is an important, but complex, task for training program leaders. Problem residents can put a strain on the program director and clinical faculty, increase the workload of other healthcare providers, and complicate patient care activities ( Williams et al., 2009). Additionally, program morale and reputation can suffer with high levels of attrition ( Gardner, Grantcharov, and Dunkin, 2018). Thus, identifying residents at risk for performance deficiencies or turnover is critical not only for trainee and program success, but also so adequate interventions can be implemented in time to avoid potential patient care issues.

We explored the extent to which SJTs could be created and used to accurately identify residents at risk for remediation, and compared its utility to the most common screening tool currently used - licensing examination scores. By following residents in a single institution over time, we found that SJTs can indeed predict those at risk for remediation. Similar to other work ( Cousans et al., 2017), our data suggests that high scores on an SJT assessment is associated with about a five times reduction in likelihood of remediation. For a specialty that remediates approximately 1 in 3 trainees at least once ( Bergen et al., 2000),and of which efforts bring substantial financial costs ( Gardner et al., 2018), these findings have considerable practical value to surgical educators. For example, the program involved in this study could administer their customized SJT to new interns to identify individuals who are more likely to struggle during clinical training and develop educational interventions accordingly. Additionally, these data contribute to the increasingly robust chain of validation reasoning that suggests SJTs may play an important role in postgraduate medical education selection ( Gardner and Dunkin, 2018a; Gardner and Dunkin, 2018b; Patterson et al. 2017).

This study failed to find support in this sample for using the SJT to identify who might leave residency prior to graduation. Although the four individuals who did leave the program within the two-year timeframe did have lower scores on the SJT, the differences among this small group were not robust enough to reach significance. Additionally, given that the majority of attrition occurs within the first few years of surgical training ( Leibrandt, Fassler, and Morris, 2004), the PGY4s and PGY5s within our sample likely represent those least at risk for turnover. Multi-institutional studies with more robust sample sizes are likely needed to properly explore these relationships.

Our findings also contribute to the burgeoning literature showing the limited value of using USMLE scores for selection ( McGaghie, Cohen, and Wayne, 2011). Although some studies have been able to link USMLE1 scores to other test scores during and after training ( Fryer et al. 2012; de Virgilio et al. 2010), many have been unable to identify a positive relationship between these scores and outcomes of interest in surgical training, including faculty evaluations ( Brothers and Wetherholt 2007; Fryer et al. 2012; McGaghie, Cohen, and Wayne, 2011; Stohl, Hueppchen, and Bienstock, 2010; Sutton et al., 2014; Zuckerman et al., 2018),professionalism metrics ( Gardner and Dunkin, 2018a), and operative experience ( Gardner and Dunkin 2018a). This lack of validity evidence, combined with concerns about how reliance on USMLE for selection may be at odds to increase diversity in surgical training ( Gardner, 2018; Gardner and Dunkin, 2018b; Higgins, 2018), suggests program leaders should explore development and adoption of tools that can be used for residency selection.

As with any study, these findings are not without limitations. First, these data include just one sample of surgery trainees within a single institution. Although the institution examined represents one of the largest general surgery training programs in the U.S., and thus includes the most robust data available from just one program, it is nonetheless a single institution study. However, given that the prevalence of remediation and attrition in this sample was lower than the national rate, these data may actually underestimate the role of the SJTs in predicting these outcomes. Further, the USMLE scores in this sample are well above the national mean. It is unknown if inclusion of a wider array of USMLE scores would have resulted in different outcomes. Finally, although the remediation data included here was retrieved from program records and thus reflect the most serious instances that were addressed, it may not fully capture all instances wherein a trainee may have received coaching from a number of different supervisors or colleagues. As with any exercise in measuring efficacy of interventions or tools, we are at the mercy of the assessment data available.

## Conclusion

These data demonstrate that SJTs can be created to effectively identify surgery residents most at risk for remediation across a two-year timeframe. These data provide an additional layer of validity evidence to support the role of SJTs in surgical education and align with other studies failing to find linkages between USMLE scores and residency performance criterion.

## Take Home Messages

• Tools are lacking to identify surgery residents most at risk for remediation and attrition
• SJTs can be created to effectively identify surgery residents most at risk for remediation across a two-year timeframe

## Notes On Contributors

Aimee K. Gardner is Co-Founder of SurgWise Consulting and Assistant Dean of Ressearch & Evaluation at Baylor College of Medicine.

Brian J. Dunkin, MD is Co-Founder of SurgWise Consulting and Professor of Surgery at Houston Methodist Hospital.

## Declarations

Both authors are co-founders of SurgWise Consulting, which provides advice on assessment to surgical training programs and organisations.
