# Supplementary material for: Making progress on identifying those who aren’t making progress: Using situational judgment tests to predict those at risk for remediation and attrition
Source: MedEdPublish (2016). 2018 Dec 7;7:275. [Version 1] doi: 10.15694/mep.2018.0000275.1 (PMC10711960; doi:10.15694/mep.2018.0000275.1)
Supplement: Peer review history

# Reviewer report: Reviewer response for version 1

**DOI:** 10.21956/mep.19734.r29569

**Published:** 2019-07-23

**Authors:** Gary D. Rogers

**Affiliations:** 1 Griffith University

This review has been migrated. The reviewer awarded 5 stars out of 5 Thank you, Aimee and Brian, for this well-written paper reporting what appears to have been a carefully-executed and important study. The positive findings, that your situational judgement tests (SJTs) predict which trainees will require remediation in relation particularly to professionalism issues in the subsequent one to two years, are truly impressive and surprising, given the concerns that have been raised about the susceptibility of SJTs to faking. This effect could be expected to be particularly prevalent among intellectually able candidates, such as those selected for surgical training. Accordingly, it would be really interesting to know more about how the SJTs were developed and how they appear to have avoided this effect – though I certainly take Barbara’s point about the risks of revealing too much detail in an environment that includes predatory commercial coaching companies. On the question of ‘what to do’ prospectively with the trainees for whom SJTs predict a high risk of professionalism breaches (apart from not selecting them!), there is some evidence pointing to the value of small-group educational interventions that promote affective domain learning through reflection on emotionally impactive clinical experiences (simulated or real) and deliberate connection with the professional values they highlight. It would be interesting to see if providing this kind of intervention to all trainees, prospectively, ‘flattens’ the effect of the SJT scores on the rate of requiring professionalism remediation.

Reviewer Expertise:

NA

No decision status is available

# Reviewer report: Reviewer response for version 1

**DOI:** 10.21956/mep.19734.r29565

**Published:** 2018-12-17

**Authors:** Balakrishnan Nair

**Affiliations:** 1 Centre for Medical Professional Development and University of Newcastle

This review has been migrated. The reviewer awarded 4 stars out of 5 It is good to see research on non -cognitive aspects of trainees. At the end of the day , unprofessional behaviour is what gets trainees into difficulty with regulatory bodies and institutions . As the authors state 1/3 of trainees in surgical training will require remediation. Some of these trainees are likely to abandon training . Any tool which may help to identify these trainees in difficulty , early enough , would be most welcome .Most of medicine is not “black and white “ . It is in the grey area where most trainees have difficulty . The script concordance tests have been popular for this reason . In this interesting study, trainees took part in Situation Judgement Testing and were followed up to assess requirements for remediation; poor test scores correlated with higher rates of remediation. Examples of SJT would have been useful , as were provided in the studies on script concordance testing . The study raises the question when to intervene: should we intervene with a low score or wait till something goes wrong ? This is a single institution study with a small sample size; it has internal validity ,but generalisation may be difficult. It will need a larger sample size and multicentre involvement to address this .

Reviewer Expertise:

NA

No decision status is available

# Reviewer report: Reviewer response for version 1

**DOI:** 10.21956/mep.19734.r29564

**Published:** 2018-12-17

**Authors:** Barbara Griffin

**Affiliations:** 1 Macquarie University

This review has been migrated. The reviewer awarded 4 stars out of 5 Given the predatory nature of the commercial coaching industry, I am not surprised that the authors did not share details of their SJT items. However, even though the small number of participants in this study precludes generalisation, I really like the robust process of item development that was described. Using data from historical cases together with SME experience to both develop and review the items may well have contributed to the significant findings. An interesting question would be whether this particular SJT relates in the same way to performance at a different institution - such information would help us know if a bespoke test is needed for each training program's specific context.

Reviewer Expertise:

NA

No decision status is available

# Reviewer report: Reviewer response for version 1

**DOI:** 10.21956/mep.19734.r29567

**Published:** 2018-12-14

**Authors:** Megan Anakin

**Affiliations:** 1 University of Otago

This review has been migrated. The reviewer awarded 4 stars out of 5 Thank you for inviting me to review this article. Like the other three reviewers, I appreciated the well-written case study that explored the use of situational judgment tests to identify at-risk learners. This article will be of interest to educators working with learners in health professional education at any stage training. For example, at my medical school, we are seeking to enhance the identification of “non-technical and non-cognitive issues” in our students earlier in the programme. We have not discussed the use and benefits of situational judgment tests as a possible solution. As reviewer Ken Masters suggested, sharing the situational judgment test constructed by the authors would have given me something concrete to present to my colleagues for discussion. Nevertheless, this article will be useful to stimulate conversation about how we might approach building an instrument fit for the purpose of our educational programme that will enable us to detect students who would benefit from more support earlier in their training.

Reviewer Expertise:

NA

No decision status is available

# Reviewer report: Reviewer response for version 1

**DOI:** 10.21956/mep.19734.r29570

**Published:** 2018-12-14

**Authors:** Ariela Marshall

**Affiliations:** 1 Mayo Clinic

This review has been migrated. The reviewer awarded 4 stars out of 5 This is an interesting and well done study and shows the potential utility of the situational judgment test for analyzing which residents may be at risk for remediation. Overall I think this makes a positive contribution to the literature regarding assessment. There are a few points which could be clarified/strengthened for future work:(1) The authors describe the creation of the SJT by a “team of MDs and PhDs” and the identification of “which competencies were critical for residents to be successful in the program.” It would be helpful to have a list of the competencies and the questions for other programs (especially in other specialties) who may wish to create their own SJTs. In addition, having this information accessible would be helpful in terms of the breakdown of the competencies – are they mostly professional, or are they technical/specialty-specific? This may help address how generalizable the findings are to non-surgical specialties(2) The authors found that most of the remediation interventions were related to deficits in professionalism, but that 33% were related to decision making and 11% to technical skills. Again, it would be useful to have lists of the exact type of deficiencies (what types of “decision making” errors – medication choice? Test ordering? Level of severity of illness? Etc) to better determine the generalizability of the study(3) It would be helpful, as another reviewer suggested, for the authors to describe “next steps” – I could see this expanding either (a) to other specialties within the same center or (b) to surgical specialties at other centers – the overall goal of course would be to all specialties at all centers, but it would be helpful to think about the next immediate goal.

Reviewer Expertise:

NA

No decision status is available

# Reviewer report: Reviewer response for version 1

**DOI:** 10.21956/mep.19734.r29568

**Published:** 2018-12-09

**Authors:** Ken Masters

**Affiliations:** 1 Sultan Qaboos University

This review has been migrated. The reviewer awarded 4 stars out of 5 A succinctly and well-written case study, clearly laying out the issues (and their importance) to be addressed, and situating the study within the literature and practical context. In particular, as the authors note, the study highlights the weakness of relying on USMLE scores for selection. I hope that the authors are planning to expand their research into other centres, in order to determine the generalisability of their SJT. On that note, it is a pity that the authors have not shared their SJT; I believe that it would of value to others working in the field.

Reviewer Expertise:

NA

No decision status is available

# Reviewer report: Reviewer response for version 1

**DOI:** 10.21956/mep.19734.r29566

**Published:** 2018-12-07

**Authors:** John Cookson

**Affiliations:** 1 University of Worcester

This review has been migrated. The reviewer awarded 4 stars out of 5 Thank you. I was interested in this as there has been much discussion of SJTs on this (Europe) side of the pond.One issue is whether the assessors can agree on the correct answers. Another is what action to take as a result of this kind of study. This paper found an association between the SJT and remediation events; most of which were about professionalism. There was no correlation with USMLE results.I think one could have predicted this; indeed if there was no association with events one would wonder if the SJT was doing anything at all. Neither is it surprising that there was no correlation with the USMLE as this and similar assessments are clearly assessing a different domain to the SJT.The problem is what to do? The authors suggest early intervention with those with a low score which certainly seems reasonable, assuming that remediation works. The wider question is whether SJTs can predict failure. We need a prediction more than a association. How well does a low score on the SJT predict subsequent problems and how good does that prediction need to be before we take action such as terminating the course. This paper suggests that that is still some way off.

Reviewer Expertise:

NA

No decision status is available
